# Stress and the HPA Axis: Balancing Homeostasis and Fertility

**DOI:** 10.3390/ijms18102224

**Published:** 2017-10-24

**Authors:** Dana N. Joseph, Shannon Whirledge

**Affiliations:** Department of Obstetrics, Gynecology, and Reproductive Sciences, Yale School of Medicine, P.O. Box 208063, New Haven, CT 06520, USA; dana.joseph@yale.edu

**Keywords:** stress, fertility, reproduction, hypothalamic-pituitary-adrenal (HPA) axis, programming, glucocorticoids

## Abstract

An organism’s reproductive fitness is sensitive to the environment, integrating cues of resource availability, ecological factors, and hazards within its habitat. Events that challenge the environment of an organism activate the central stress response system, which is primarily mediated by the hypothalamic–pituitary–adrenal (HPA) axis. The regulatory functions of the HPA axis govern the cardiovascular and metabolic system, immune functions, behavior, and reproduction. Activation of the HPA axis by various stressors primarily inhibits reproductive function and is able to alter fetal development, imparting a biological record of stress experienced in utero. Clinical studies and experimental data indicate that stress signaling can mediate these effects through direct actions in the brain, gonads, and embryonic tissues. This review focuses on the mechanisms by which stress activation of the HPA axis impacts fertility and fetal development.

## 1. Introduction

Stress is commonly defined as a state of real or perceived threat to homeostasis that may challenge an organism’s well-being [1]. In order to restore homeostatic conditions, organisms activate a complex range of responses involving the endocrine, nervous, and immune systems, collectively known as the stress response [2]. The stress response serves to prioritize survival over less essential physiological functions, including growth and reproduction. The hypothalamic–pituitary–adrenal (HPA) axis, comprised of the hypothalamus, pituitary gland, and adrenal glands, regulates the body’s adaptive response to stress [1]. Activation of the HPA axis triggers neurons in the paraventricular nucleus (PVN) of the hypothalamus to release corticotropin-releasing hormone (CRH) and arginine vasopressin (AVP), which stimulate the anterior pituitary gland to produce and secrete adrenocorticotropic hormone (ACTH) [3]. In response, ACTH induces the synthesis and secretion of glucocorticoids (cortisol in humans and corticosterone in rodents), mineralocorticoids (aldosterone), and adrenal androgens, which are released from the adrenal cortex into the blood circulation [3,4]. Rising levels of cortisol inhibit further release of CRH and ACTH in a classic endocrine negative feedback loop, which enables the HPA axis to return to a physiological state following acute activation.

As part of the physiological adaptation to stress, the HPA axis mediates the functions of the hypothalamic–adrenal–gonadal (HPG) axis, which is responsible for the maturation of the reproductive organs and the reproductive competence of an organism. The HPG axis controls the reproductive system through endocrine signaling, originating with hypothalamic secretion of gonadotropin-releasing hormone (GnRH). GnRH stimulates the gonadotroph cells of pituitary to synthesize and release follicle stimulating hormone (FSH) and luteinizing hormone (LH). In turn, FSH and LH act on the ovary to regulate oocyte maturation, ovulation, and steroid hormone production [5]. Activins, inhibins, and ovarian produced steroid hormones (estradiol and progesterone) feedback to regulate the secretion of the gonadotrophins [6]. Stress signaling impacts all levels of the HPG axis [7]. For example, high levels of glucocorticoids have inhibitory effects on the GnRH neurons, the pituitary gonadotrophs, and the gonads [7,8]. The purpose of this review is to briefly introduce the signaling mechanisms of stress hormones, summarize recent advances in our understanding of the effects of stress on fertility, and discuss current evidence that describes the long-term effects of stress experienced during in utero development.

## 2. Mechanisms of Hormone Signaling

Glucocorticoids, named for their effects on glucose metabolism, are critical regulators of metabolic homeostasis, the cardiovascular system, cell proliferation and survival, growth, cognition and behavior, immune function, and reproduction [9]. Due to their potent anti-inflammatory and immunosuppressant actions, glucocorticoids are frequently utilized for clinical applications and in over-the-counter medications. In fact, it is estimated that 1.2% of the U.S. population, or approximately 3.6 million people, use therapeutic glucocorticoids [10]. The pharmacological and cellular actions of glucocorticoids are mediated by the glucocorticoid receptor (GR), a member of the nuclear receptor superfamily of ligand-dependent transcription factors. Similar to the other members of the nuclear receptor superfamily, GR has a modular structure comprised of three distinct functional domains: an amino-terminal transactivation domain, a central DNA binding domain, and a carboxy-terminal ligand-binding domain. Between the DNA-binding domain and the ligand-binding domain lies a flexible hinge region that provides structural flexibility for genomic interactions and contains a nuclear localization signal.

GR is able to actively shuttle between the cytoplasm and nucleus in response to ligand binding. In the absence of the endogenous ligand or synthetic glucocorticoids (e.g., dexamethasone, betamethasone, hydrocortisone, prednisone), GR resides predominantly in the cytoplasm as part of a large multi-protein complex that includes chaperone proteins (heat shock protein (hsp) 90, hsp70, and p23) and immunophilins of the FK506 family (FK506-binding protein (FKBP) 51 and FKBP52) [11,12]. Ligand binding induces a conformational change that promotes nuclear accumulation of GR and allows interactions with specific genomic loci and other transcription regulators. GR binding at DNA sequences termed GR response elements (GREs) is the most frequently described GR–DNA interaction [13]. The consensus GRE sequence is two hexameric half-sites separated by a three-nucleotide spacer (AGAACAnnnTGTTCT) [14]. GR bound to DNA promotes remodeling of the chromatin, prompts the recruitment of co-regulators, and stimulates initiation of transcription. GR can also bind negative GREs (nGREs) that have a less well-defined consensus sequence (CTCC(n)_0-2_GGAGA) [15]. The binding of GR to nGREs leads to transcriptional repression through the recruitment of co-repressors. GR is also able to mediate gene transcription through interactions with other transcription factors, either with or without contacting DNA. The nature and magnitude of the cell’s response to glucocorticoids is dependent on the ligand dose and type, post-translational modifications to GR, relative abundance of co-regulators, the chromatin environment, and the GR-bound DNA sequence [16].

Both glucocorticoids and mineralocorticoids bind the mineralocorticoid receptor (MR), although receptor occupancy is determined by the 11β-hydroxysteroid dehydrogenase (HSD) enzymes, 11β-HSD1, and 11β-HSD2. Glucocorticoids have a 10-fold higher affinity for MR, but in tissues that express both MR and 11β-HSD2, cortisol is catabolized into an inactive metabolite allowing aldosterone to bind MR [17]. Like GR, MR also belongs to the nuclear receptor superfamily of ligand-dependent transcription factors and regulates gene transcription by transactivation or transrepression in response to its association with different MR-interacting proteins [18]. The tissue distribution of MR is more restricted than GR, found primarily in the kidney, colon, heart, certain regions of the brain, and various tissues of the reproductive tract (www.proteinatlas.org). Under physiological conditions, MR functions to regulate ion and fluid transport to maintain osmotic and hemodynamic homeostasis. However, MR signaling is also believed to be necessary for the cognitive response to stress, and mediating memory storage of the inciting event and facilitating behavior adaptation [19].

## 3. The Impacts of Stress throughout the HPG Axis

Stress-mediated inhibition of GnRH release has been demonstrated in several species, including fish, birds, rodents, livestock, and humans [20,21,22]. In sheep, acute stress (isolation, transportation, injection of endotoxin, experimentally induced hypoglycemia, or infusion of cortisol) suppresses GnRH and gonadotropin release [23]. These effects are mediated through suppression of GnRH and GnRH receptor (GnRHR) synthesis, disruption in pituitary release of LH, and enhanced function of the gonadotropin-inhibitory hormone (GnIH; mammalian ortholog gene *Rfrp3*) neurons [21,23,24,25]. Restraint stress in mice and rats leads to increased expression of hypothalamic *Rfrp3* associated with elevated circulating levels of corticosterone and decreased functions of the HPG axis [26,27]. Interestingly, silencing *Rfrp3* expression utilizing a cytomegalovirus (CMV) promoter-driven viral vector in female rats blocked stress-induced reproductive dysfunction [27]. The effects of immobilization stress on female mice have also been shown to reduce GnRH-mediated LH synthesis and secretion [28]. Chromatin immunoprecipitation assays in a mouse pituitary gonadotroph cell line indicate that GR is recruited to the *LHβ* promoter in a GnRH-dependent manner. Inhibition of the LH surge has also been documented in mice following psychosocial stress [29]. Severe illness and traumatic brain injury in post-menopausal women have been associated with a reduction of LH and FSH levels [30,31]. These data further support the inhibitory effects of stress on reproductive function. The levels of LH secretion have also been studied in response to glucocorticoid treatment in rats. It has been demonstrated that secretion of LH and FSH by female rat pituitary cells exhibits differential responsiveness to treatment with glucocorticoids in vitro [32]. Basal secretion of LH was inhibited, whereas basal secretion of FSH was enhanced. Other studies have highlighted a negative impact of a basal cycle high FSH:LH ratio (and possibly low LH levels) on follicular development and oocyte quality [33]. In vivo, mice exposed to chronic corticosterone exhibit significantly decreased LH levels, associated with disrupted neuronal activation in the hypothalamus and disrupted gene expression in the pituitary gonadotroph cells [34].

The ability of CRH to disrupt reproductive functions, particularly in the absence of circulating steroids of adrenal and/or gonadal origin, has also been examined. In a study consisting of acute administration of ovine CRH into the lateral ventricle of gonadectomized, adrenalectomized female rats, a rapid and prolonged dose-related inhibition of LH (but not FSH) secretion was exhibited [35]. Additionally, CRH injected daily to female rats during the first 12 days after mating caused a 40% disruption of pregnancy [35]. These results suggest that CRH exerts negative effects on reproductive functions. Restraint stress in rats at proestrus acutely raised plasma concentrations of LH and FSH, which was blocked by CRH Receptor 1 (CRH-R1) inhibitors [36]. The stimulatory effects of stress on the HPG axis in this paradigm may reflect the timing of stress exposure. The effect of exogenous CRH on pulsatile gonadotropin secretion and the role of endogenous opioid peptides in this phenomenon have been studied in women. CRH infusion during the mid-luteal phase induced a significant decrease in plasma LH and FSH levels [37]. Plasma LH and FSH concentrations returned to basal levels shortly after the end of the CRH infusion, and it was observed that CRH infusion did not alter the gonadotropin response to GnRH [37]. The disruptive effect of stress on reproductive function may be dependent on decreased gonadotropin secretion induced by elevated endogenous CRH levels.

### 3.1. Effects in Female Reproductive Tract Organs

Stress-induced levels of glucocorticoids have been shown to impair oocyte competence. Pigs exposed to chronic heat stress demonstrate altered ovarian expression of the genes that encode the steroidogenic enzymes and increased signaling through the insulin-mediated phosphoinositide 3-Kinase (PI3K) pathway [38]. Conditions associated with increased insulin signaling (such as obesity and polycystic ovary syndrome) are also associated with reproductive dysfunction [39]. Heat stress can also change the follicular fluid composition, altering the oocyte microenvironment [40,41]. In mice, the degree of damage to oocyte competence was dependent on the severity of restraint stress, with greater effects found after long-term stress [42]. Exogenous exposure to glucocorticoids in female rats led to a decrease in the levels of growth factors and changes to the ratio of estrogen to progesterone [43]. Corticosterone exposure induced the apoptosis of ovarian epithelial cells, leading to a decline in growth factor levels and estrogen to progesterone ratio and an increase in FasL in the follicular fluid, impairing oocyte developmental potential. The effect of glucocorticoids on human oocytes has also been evaluated [44]. The study correlated levels of intra-follicular glucocorticoids to oocyte maturation and successful fertilization in oocytes recovered from women undergoing in vitro fertilization. The authors reported that follicular fluid from follicles whose oocytes were not fertilized had levels of cortisol significantly higher than the levels in follicular fluid from follicles containing successfully fertilized oocytes [44]. This suggests that high levels of glucocorticoids negatively influence the ability of an oocyte to become fertilized.

**Figure 1:**
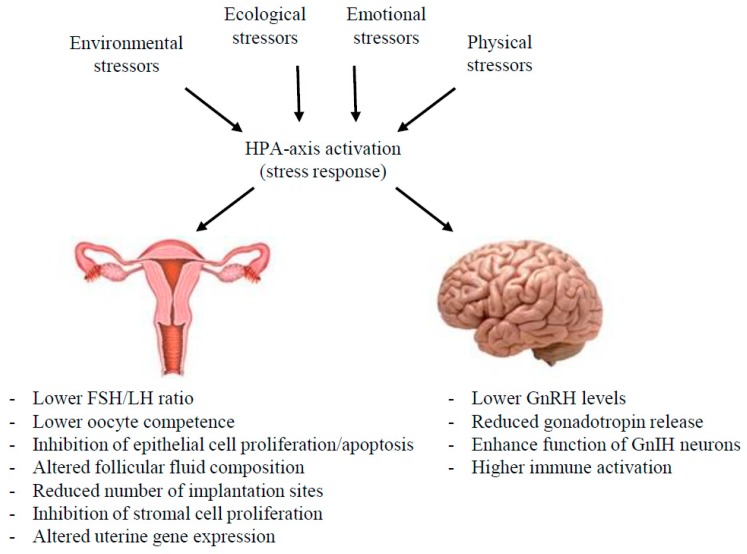
HPA axis activation by various stressors alters the activity of the HPG axis. HPA-axis: hypothalamic-pituitary-adrenal axis, FSH: follicle stimulating hormone, LH: luteinzing hormone, GnRH: gonadotropin-releasing hormone, GnIH: gonadotropin-inhibitory hormone (*Rfrp3*).

Restraint stress impairs fertility in female mice through several mechanisms (Figure 1). Mated female mice with elevated levels of glucocorticoids in response to restraint stress displayed pronounced apoptosis in the oviduct, poor embryo development, and fewer blastocysts per mouse [45]. Restraint stress in pregnant mice significantly reduced the pregnancy rate and average litter size of mice as compared to controls, attributed in part to reduced number of implantation sites [46]. Decreased endometrial cell proliferation, vascular endothelial growth factor expression, and microvessel density were found in mated female mice exposed to restraint stress, which may provide some insight into the mechanisms by which restraint stress negatively influences the uterine environment [47]. Auditory stress on female mice has also been reported to have adverse effects on uterine receptivity [48]. Auditory stress was shown to cause an increase in anxiety-like behavior and was associated with higher rates of resorbed embryos and reduced litter size. These results suggest that different types of stress inputs can negatively influence uterus receptivity.

Studies with exogenous exposure paradigms have further demonstrated the uterine effects of glucocorticoids. In rats, administration of cortisol blocked estrogen-induced changes in uterine weight and Na^+^, Cl^−^, and K^+^ concentrations [49]. Recent studies have shown that high Na^+^/Cl^−^ and low K^+^ levels in utero can be detrimental to embryo attachment through the establishment of an unfit, unreceptive uterine environment [50]. Dexamethasone also blocks estrogen-induced uterine growth and proliferation in mice [51]. Dexamethasone treatment uniquely regulates the expression of genes in the mouse uterus and in human uterine cell lines with functions that are related to signaling, embryonic development, and cellular growth and proliferation [52,53,54] Epithelial cell proliferation prior to implantation is critical to establishing a receptive uterus. In the mouse uterus, dexamethasone blocks estrogen-mediated induction of insulin-like growth factor-I (*Igf-1*), a key growth factor stimulating epithelial cell proliferation [55,56,57]. Following implantation, the uterine stroma proliferates and differentiates into secretory decidual cells. Human endometrial stromal cells decidualized ex vivo demonstrate distinct GR and MR transcriptional networks, suggesting stress signaling through release of cortisol and aldosterone may impact uterine physiology through independent mechanisms [58].

### 3.2. The Placenta and Parturition

Throughout the second and third trimesters of pregnancy, human placental syncytiotrophoblasts secrete substantial quantities of CRH, with peak levels occurring at parturition [59]. As a result, pregnant women have CRH concentrations that are 1000 to 10,000 times greater than that of non-pregnant individuals [60]. Although a majority of CRH is bound by CRH-binding protein (CRH-BP) in an inactive form, the surge in CRH during the third trimester is not accompanied by a coordinate increase in CRH-BP levels, resulting in higher concentrations of active CRH in circulation [61]. It has been hypothesized that the increasing levels of CRH may serve as a “placental clock,” a mechanism that is responsible for the duration of gestation and the onset of a procontractile phenotype at the initiation of labor [60]. CRH receptors have been identified in the human myometrium [62]. The direct actions of CRH on myometrial contractility involve distinct CRH receptor isoforms. The R1 subtype mediates signaling pathways that inhibit phosphorylation of contractile proteins, thereby promoting smooth muscle relaxation [63]. However, expression of CRH-R1 decreases in the upper segment of the uterus during labor, which becomes highly contractile [64]. By contrast, CRH-R2 expression increases in the upper segment of the uterus during labor, although its function is still unclear [65]. In support of a procontractile phenotype, CRH increased the expression of the contraction associated gene connexin-43 (*Cx43*) in myometrial smooth muscle cells [66]. CRH also stimulated the production of chemokines and cytokines in cultured uterine smooth muscle cells, which have been shown to activate the uterus through the induction of an inflammatory response and the recruitment of immune cells [67,68]. The transition from a quiescent to a contractile myometrium is also mediated by a rise in estrogen levels near the end of gestation. Elevated CRH, via cortisol, mediated the production of estrogen by increasing aromatase expression in human placental syncytiotrophoblasts [69].

Interestingly, CRH produced in the placenta can stimulate ACTH production in the pituitary, leading to elevated levels of glucocorticoids. In turn, glucocorticoids have been shown to stimulate placental CRH synthesis and secretion in cultured human cytotrophoblasts [70]. This reflects a positive feedback loop that could amplify the effects of stress-induced levels of cortisol leading to the initiation of preterm labor.

Preterm birth is the leading cause of infant mortality and morbidity, and despite substantial resources devoted to reducing the rate of prematurity, the prevalence in the U.S. population has not decreased in the last 25 years [71]. In fact, the March of Dimes reports that preterm birth rates have recently risen in the USA (www.marchofdimes.org). Although, the etiology of preterm birth likely involves numerous factors, maternal stress has been identified as a potential casual factor for preterm birth [72]. In a study of 9350 U.S. mother–infant pairs, a stressful event reported prior to conception was associated with a 4-fold increased risk for preterm birth [73]. A population-based study in Denmark also described an association between a stressful life event occurring within the six months prior to conception and an increased risk for preterm delivery [74]. In a small study of African-American women, childhood stress, independent of stress in adulthood, was associated with early birth timing [75]. Women experiencing high levels of psychosocial stress during pregnancy are at significantly increased risk for shortened gestation and preterm delivery, even after accounting for the effects of established risk factors such as sociodemographic, biophysical, biomedical, and behavioral [76].

Although several studies have shown an association between stress and preterm labor, stress experienced prior to conception or during pregnancy does not necessarily predict preterm birth. The mechanisms underlying the association between stress and preterm delivery are not clear. Some studies have demonstrated elevated HPA activity in these women, including elevated cortisol and CRH levels [77,78,79]. Naturally, cortisol levels rise toward the end of gestation and at the onset of labor. However, stress-induced levels of cortisol may inappropriately stimulate the signaling mechanisms contributing to initiation of labor. Apoptosis of the amnion epithelial layer precedes fetal membrane rupture, an initiating factor for both term and preterm birth. Ex vivo culture of human amnion epithelial cells with cortisol-induced cell death through the extrinsic apoptotic pathway [80]. Cortisol also decreased the abundance of collagen proteins and lysyl oxidase, a collagen cross-linking enzyme, in amnion cells through lysosome-mediated autophagy [81]. Collagen content determines the tensile strength of the amnion and reduced collagen content has been demonstrated in women with preterm rupture of membranes [82]. The degenerative actions of glucocorticoids in amnion cells may contribute to premature rupture of fetal membranes leading to preterm labor. Production of prostaglandin E2 (PGE2) by the amnion increases at the onset of labor, which contributes to myometrial contractions and the breakdown of the physical integrity of the cervix. Glucocorticoid treatment in cultured term human amnion cells significantly enhanced PGE2 expression, which has also been demonstrated in domestic animal species [83,84]. Generally synthetic glucocorticoids given to women at risk of preterm labor to induce fetal lung maturation do not cause labor, although some studies have reported the initiation of labor following glucocorticoid injection in women post-term [85,86]. These discrepancies may reflect differences in the timing of exposure, particularly in relationship to other signals that coordinate labor.

### 3.3. Other Markers of the Stress Response

Salivary α-amylase (sAA) is secreted in response to stress-activation of the sympathoadrenal medullar system and has been identified as a novel biomarker of the physiological and psychosocial stress response [87]. It was found that women in the highest tertile of α-amylase exhibited a significant reduction in fecundity, and found no association between salivary cortisol and fecundability [88]. This study concluded that stress (as measured by increased sAA) is associated with lower fecundity among affected women. Moreover, stress significantly reduced the probability of conception each day during the fertile window [89]. While these studies certainly do not give a definitive answer regarding causation, they provide further evidence of the independent adverse role that stress might play.

## 4. Stress Impact on Programming

**Figure 2:**
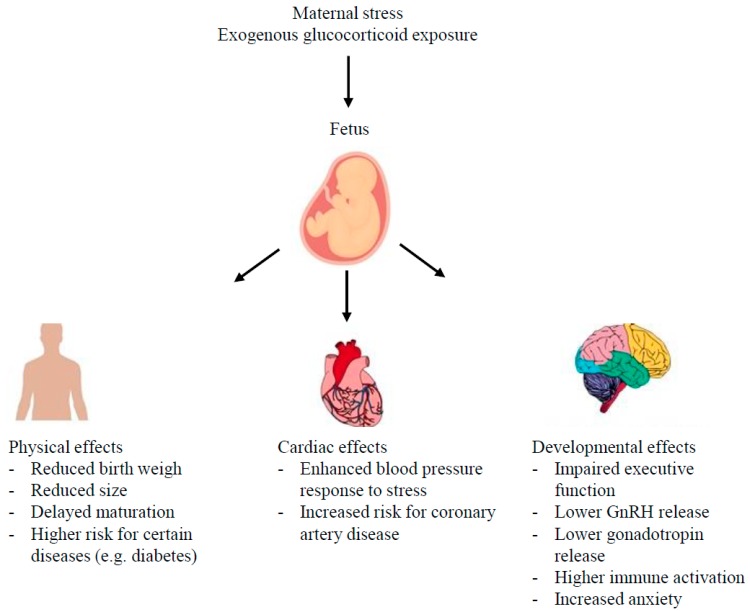
High levels of glucocorticoids experienced in utero through stress or exogenous administration can cause effects in the organ systems of offspring.

Glucocorticoids are crucial during fetal development for the functional maturation of the respiratory system [90,91,92] and are currently utilized as the standard of care for women at high risk for preterm delivery to prevent respiratory distress syndrome [93]. The wide-spread use of antenatal glucocorticoid therapy has significantly increased the survival rate of premature infants. While glucocorticoid treatment is necessary for survival, increased glucocorticoid exposure, either by stress or by exogenous treatment, can lead to long-lasting effects in offspring (Figure 2) [7,94]. Given their essential actions during fetal growth and development, excess glucocorticoids likely represent a signaling pathway by which poor environmental conditions are signaled from the mother to the fetus, triggering changes in offspring growth and permanently affecting tissue and organ function [94]. Several studies conducted in a range of species have shown that maternal stress or prenatal exposure to high levels of glucocorticoids reduces birth weight. These offspring are also at an increased risk of cardio-metabolic disease, HPA axis perturbations, and affective disorders in later life [95]. Moreover, recent studies indicate that the effects of antenatal glucocorticoid exposure may persist for several generations [96].

Studies have shown that the offspring of women who were pregnant at the time of disasters have offspring with an increased risk for developmental disorders. During the Dutch famine (1944–1945), babies exposed to maternal famine during late or mid-gestation were lighter, shorter, thinner and had a smaller head circumference than babies that had not been exposed to the famine [97]. These offspring also had an increased risk of other health problems, including a higher rate of coronary artery disease and type 2 diabetes, enhanced blood pressure response to stress, poor cognitive performance, and an increased risk of schizophrenia and depression [98]. Genome-wide analysis revealed several regions of DNA which were differentially methylated in offspring born from mothers of the Dutch famine, including genes related to growth and metabolism, which may contribute to the phenotypes reported [99]. In a separate study, differential methylation of *IGF-1* in response to stress was associated with low birth weight in an African population [100]. Women who were directly exposed to the World Trade Center collapse in 2001 (either by being present or near the building while pregnant) and developed post-traumatic stress disorder (PTSD) gave birth to offspring that had lower birth weight and cortisol concentrations than offspring who were not exposed to maternal PTSD [101,102]. The impact of exposure to natural disasters during pregnancy has also been associated with educational outcomes of the offspring. Children exposed to natural disasters like hurricanes, tornadoes, and flooding prenatally have lower scores on third grade standardized tests [103]. The offspring of women who were pregnant during the January 1998 ice storm crisis in Québec, Canada that resulted in power losses for 3 million people for up to 40 days had lower full Scale IQs, verbal IQs, and language abilities compared to children exposed to low or moderate levels of objective prenatal maternal stress [104]. It is important to note that IQ tests alone do not accurately measure cognition, but these studies suggest that women who experience stress during pregnancy may foster an adverse prenatal environment that leads to deficiencies in offspring [105].

Maternal stress in pregnancy can shape childhood outcomes in a range of developmental domains including temperament, cognition, language skills, and motor functioning [106,107]. High levels of maternal pregnancy-specific anxiety was associated with increased negative temperament in children [108]. Elevated levels of maternal pregnancy-specific anxiety early in pregnancy were also independently associated with lower scores on the Bayley Scales of Infant Development (BSID) at 12 months [109]. Exposure to elevated concentrations of cortisol early in gestation has also been associated with a slower rate of development over the first postnatal year and lower scores on the mental development index of BSID at 12 months, while elevated levels of maternal cortisol late in gestation were associated with accelerated development over the first year and higher scores on the BSID at 12 months [109]. These data suggest that pregnancy-specific anxiety and the timing of maternal cortisol exposure influence fetal development. Maternal stress in other mammals has also been linked to adapted fetal outcomes. For example, heat stress during gestation in pigs altered back-fat depth and elevated circulating insulin in offspring later in life [110]. In utero exposure to heat stress also influenced the thermoregulation of offspring [111]. Pigs developed during in utero heat stress experienced elevated body temperature regardless of postnatal environmental conditions [111,112]. The effects of fetal exposure to synthetic glucocorticoids on children’s susceptibility to postnatal sociodemographic adversity have also been studied. Children exposed to both prenatal stress hormones and sociodemographic adversity showed impaired performance on standardized tests of long-term memory function, these results being independent of maternal intelligence and concurrent maternal depression [113]. These findings suggest that exposure to synthetic stress hormones may be associated with increased susceptibility to subsequent adversity with consequences for cognitive functioning that persist for six to 10 years after birth [113].

### Sex Differences in the Response to Stress

At birth, the placenta and cord blood from babies born to mothers of lower socioeconomic status, which has been associated with psychological stress, cortisol dysregulation, and poor environmental conditions, exhibit a transcriptional profile of higher immune activation and delayed maturation, illustrating that the in utero response to stress results in molecular signaling changes [114,115,116]. Furthermore, evidence suggests that male and female offspring differ in their responses to prenatal stress [117]. The female placenta was shown to respond to changes in glucocorticoid concentration with changes in cortisol metabolism, placental cytokine expression, IGF axis signaling, adrenal function and growth, while the male placenta appears glucocorticoid resistant, since pathways typically responsive to cortisol such as cytokine expression, the IGF axis, adrenal function, and growth remained unaffected [118]. The male response was associated with a greater risk of either intrauterine growth restriction, preterm delivery, or death in utero, while the female response involved adaptations that resulted in decreases in growth in order to promote survival [118].

The female placenta may facilitate higher glucocorticoid exposure, as the activity of the 11β-hydroxysteroid dehydrogenase enzymes (11β-HSD1 and 11β-HSD2) responsible for cortisol metabolism was found to be altered in the placenta of females [117,119]. Moreover, isoforms of GR, which exhibit unique transcriptional profiles, are differentially expressed in the placenta of male and female mice in response to maternal dexamethasone exposure [120,121]. Studies in the murine placenta have reported sex-specific differences in the whole genome transcriptional response to dexamethasone, where the placenta from male offspring preferentially expressed genes related to inflammation and the immune response [122]. Studies also show that prenatal maternal stress can differentially affect outcomes for each sex beyond the neonatal period [106]. The effects of female fetal exposure to stress persists into preadolescence, as reflected in increased levels of anxiety, impaired executive function, and neurological markers associated with these behaviors [123]. Additionally, females, but not males, exposed to elevated levels of maternal cortisol early in pregnancy had significantly enlarged amygdala and increased levels of anxiety in childhood [123]. Maternal stress, as measured by sAA, has also been associated with negative emotionality in females but not male offspring [124]. These data show that early-life exposure leads to sexually dimorphic outcomes in fetal programming.

## 5. Conclusions

While basal glucocorticoid levels are necessary for fertility and fetal survival, upregulation due to acute HPA axis activation or exogenous treatment can have detrimental effects on fertility and fetal outcomes. Elevated HPA activity is associated with altered functions of the hypothalamus, pituitary, and gonads. Moreover, the offspring of stressed mothers can present with abnormalities including reduced birth weight, higher levels of anxiety, higher levels of HPA axis function, and are at an increased risk of developing various physiological diseases. Reproductive dysfunction as a result of stress or exogenous glucocorticoid exposure has been documented in several tissues of the HPG axis, and the mechanisms have been examined in some of those tissues. However, it is still not well understood whether the phenotypes documented arise from direct actions on target tissues or the endocrine effects of stress signaling. Moreover, the role of the other adrenally produced hormones and MR signaling has not been well defined in the reproductive system. Other issues that concerns this area of research include trying to isolate confounding variables, as stress signaling is often associated with other types of disorders [125]. Studying the interplay between the neuroendocrine and the HPG axis, particularly to ascertain how chronic stress modifies the signaling pathways in the tissues of the reproductive system, would provide insight into the specific mechanisms of stress-mediated dysfunction.
